# Preliminary Use and Outcome Data of a Digital Home Exercise Program for Back, Hip, and Knee Pain: Retrospective Observational Study With a Time Series and Matched Analysis

**DOI:** 10.2196/38649

**Published:** 2022-12-02

**Authors:** Gisbert Wilhelm Teepe, Tobias Kowatsch, Felix Patricius Hans, Leo Benning

**Affiliations:** 1 Center for Digital Health Interventions ETH Zürich Zürich Switzerland; 2 Institute for Implementation Science in Health Care University of Zürich Zürich Switzerland; 3 School of Medicine University of St.Gallen St.Gallen Switzerland; 4 University Emergency Center, Medical Center - University of Freiburg Freiburg Germany; 5 Faculty of Medicine University of Freiburg Freiburg Germany; 6 Vivira Health Lab GmbH Berlin Germany

**Keywords:** digital health, home exercise, musculoskeletal conditions, digital intervention, exercise, physical activity, smartphone, pain, management, back pain, hip pain, knee pain, mobility, intervention

## Abstract

**Background:**

Musculoskeletal conditions are among the main contributors to the global burden of disease. International guidelines consider patient education and movement exercises as the preferred therapeutic option for unspecific and degenerative musculoskeletal conditions. Innovative and decentralized therapeutic means are required to provide access to and availability of such care to meet the increasing therapeutic demand for this spectrum of conditions.

**Objective:**

This retrospective observational study of preliminary use and outcome data explores the clinical outcomes of Vivira (hereafter referred to as “program”), a smartphone-based program for unspecific and degenerative pain in the back, hip, and knee before it received regulatory approval for use in the German statutory health insurance system.

**Methods:**

An incomplete matched block design was employed to assess pain score changes over the intended 12-week duration of the program. Post hoc analyses were performed. In addition, a matched comparison of self-reported functional scores and adherence rates is presented.

**Results:**

A total of 2517 participants met the inclusion criteria and provided sufficient data to be included in the analyses. Overall, initial self-reported pain scores decreased significantly from an average of 5.19 out of 10 (SD 1.96) to an average of 3.35 out of 10 (SD 2.38) after 12 weeks. Post hoc analyses indicate a particularly emphasized pain score reduction over the early use phases. Additionally, participants with back pain showed significant improvements in strength and mobility scores, whereas participants with hip or knee pain demonstrated significant improvements in their coordination scores. Across all pain areas and pain durations, a high yet expected attrition rate could be observed.

**Conclusions:**

This observational study provides the first insights into the clinical outcomes of an exercise program for unspecific and degenerative back, hip, and knee pain. Furthermore, it demonstrates a potential secondary benefit of improved functionality (ie, strength, mobility, coordination). However, as this study lacks confirmatory power, further research is required to substantiate the clinical outcomes of the program assessed.

**Trial Registration:**

German Clinical Trials Register DRKS00021785; https://drks.de/search/en/trial/DRKS00021785

## Introduction

Musculoskeletal conditions (MSCs) are among the most important contributors to the global burden of disease [1]. As the most prevalent disorders among working populations, they not only contribute greatly to direct but also to indirect health care costs [2]. At the same time, the access to and availability of adequate therapeutic means for the MSC spectrum remain challenging [3]. Yet, it has repeatedly been shown that different kinds of physical activity (PA), especially structured exercise programs, effectively address certain kinds of MSCs. This particularly applies to unspecific and degenerative musculoskeletal pain (MSP) [4-6].

PA has been studied in numerous digital health intervention studies far beyond the clinical spectrum of MSC. A recent meta-analysis by Mönninghoff et al [7] showed that PA (measured as walking standardized mean difference, moderate-to-vigorous physical activity standardized mean difference, total physical activity standardized mean difference, and energy expenditure standardized mean difference) could be improved at the end of the intervention. Nevertheless, effect sizes decreased over time in the 33 studies reporting short-term and in the 8 studies reporting long-term (ie, postintervention) follow-up. Additionally, effect sizes were moderated by the study population, with higher effect sizes in sick and at-risk populations (ie, sedentary, older, overweight), indicating the higher impact of digital health interventions for such populations and the necessity to evaluate digital health interventions in respective clinical settings thoroughly.

Focusing on PA changes in patients with chronic MSP, a meta-analysis by Oliveira et al [8] found that PA interventions compared to no or minimal interventions in patients with chronic muscular pain showed no significant improvement over short-term, intermediate, or long-term follow-up. Most of these studies delivered their intervention in a nondigital blended approach consisting of an instructional part in a face-to-face setting and an exercise part to be completed independently at home. In comparison to the review by Mönninghoff et al [7], only 1 study included in the review by Oliveira et al [8] used a digital component (web-based PA intervention over 9 weeks that incorporated a baseline test; goal setting, time-contingent physical activity objectives; and text messaging to promote physical activity). However, the relatively low number of studies included (8 randomized controlled trials) in Olivera et al [8] may substantially limit this finding and highlights the need for further studies investigating the effect of interventions on PA in MSC patients and the added effect of using digital components in interventions.

To address the outlined challenge, this study presents preliminary use data of Vivira, a smartphone-based program for unspecific and degenerative pain in the back, hip, and knee. It also demonstrates early data on self-reported pain score reductions and functional improvements, as well as data on adherence to the program.

## Methods

### Study Design

This study presents observational data on the primary outcome of overall pain score reduction and the secondary outcomes of reporting interval-specific and stratum-specific pain score reductions, functional improvement, and retention to the program. Clinical outcomes are collected with self-reported pain scores, assessed with a verbal-numerical rating scale (VNRS), which has been established to be a reliable [9] and valid instrument [10] to capture pain score intensity as a participant-reported outcome measure. The primary hypothesis test for assessing pain score changes is a nonparametric, 2-sided Skillings-Mack test, outlined elsewhere in detail [11]. In brief, it allows the analysis of an unbalanced and incomplete block design with relevant missing data by design or random. The functional assessment is developed based on established orthopedic functional tests and employs the principles of functional regional interdependence [12-15]. To enable a participant-directed self-assessment, these tests are presented with audiovisual guidance. Results are entered on a binary scale (ie, the test could be completed, or the test could not be completed). In this study, through expert consensus of a panel of orthopedic surgeons and physical therapists, the weighted transformation of the functional tests was performed to compute discrete functional scores. A Wilcoxon signed-rank test, a Kruskal-Wallis test, and a 1-way ANOVA were used for secondary analyses of pain and functional scores. Distributions were assessed using the Bartlett test.

Corrections for familywise errors were performed using the Bonferroni procedure. Retention was assessed based on whether participants started to use the program (ie, completed at least 1 exercise) and submitted a complete pain assessment at predefined thresholds (2 weeks, 4 weeks, 8 weeks, and 12 weeks). Participants were enrolled through a self-selection process of voucher-based mass campaigns and early self-pay subscriptions between January 9, 2018, and June 15, 2020. Inclusion criteria are outlined in Textbox 1. Additional data on the pain duration (ie, acute, <6 weeks; subacute, 6-12 weeks; chronic, ≥12 weeks [16]) were collected to allow stratum-specific analyses.

**Inclusion criteria for this study.**

- Age ≥18 years
- Report of any applicable pain area (ie, upper back, lower back, hip, or knee)
- Initial pain score assessed with the verbal-numerical rating scale (VNRS) >0/10
- Completion of at least 1 exercise during the study period

### Ethics Approval

This study received approval from the ethics committee of the Landesärztekammer Baden-Württemberg (state physician chamber of Baden-Württemberg) under the reference F-2020-075 and is registered with the German Clinical Trials Register DRKS under the reference DRKS00021785.

### Exercise Program and Composition of Exercise Regimes

**Figure 1:**
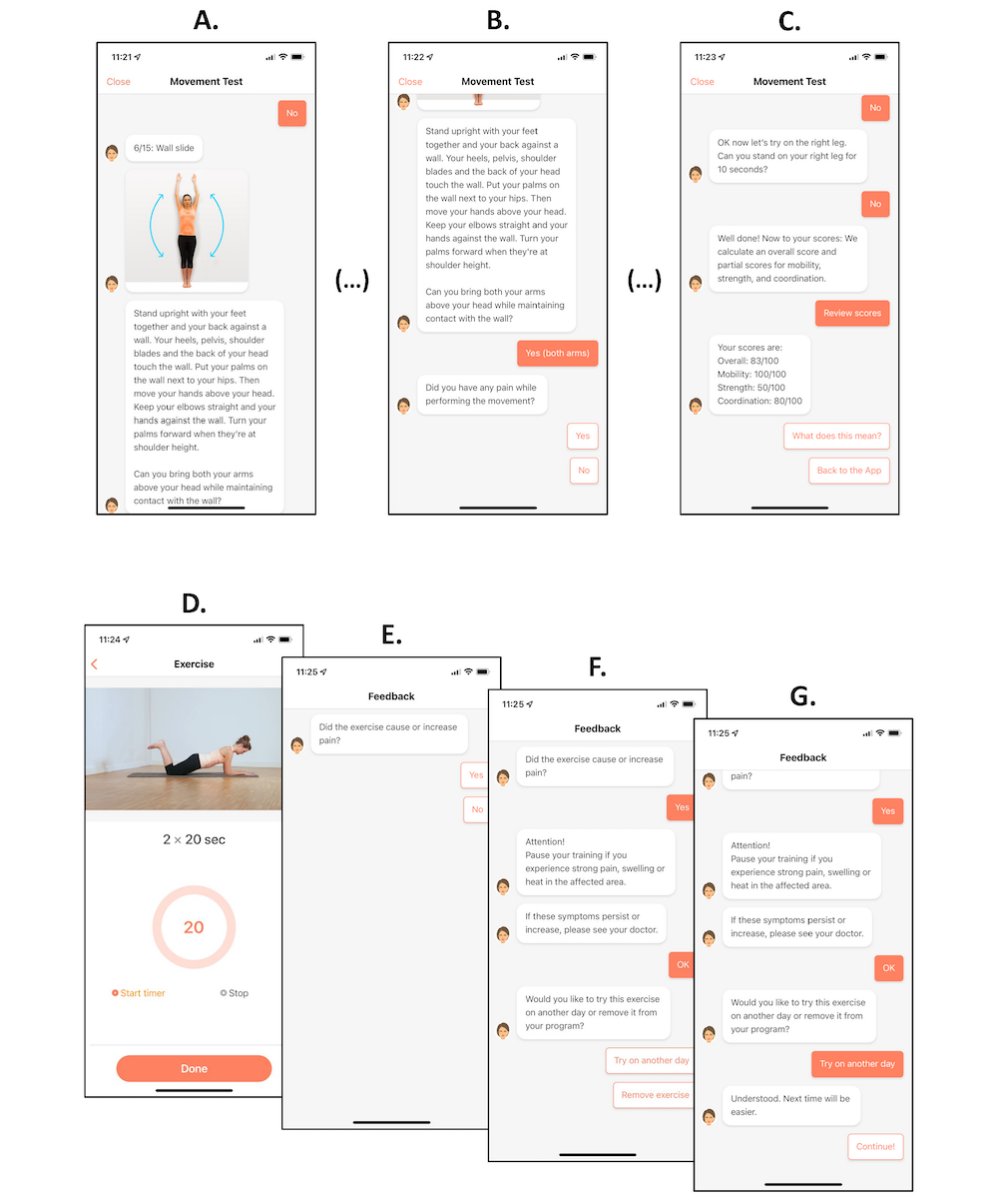
Examples of the baseline assessment and the progression module. A. Patients are prompted to perform certain exercises with visual and text-based aids. B. Pain and movement limitations are assessed. C. A baseline functional score is computed and used as the intraindividual benchmark for further assessments. D. After the completion of any exercise, patients are required to report any pain sensations. E. If pain is reported, a warning is issued. F. Patients can select whether they want to exclude the exercise from their training program, or whether they want to regress to an easier version of the same exercise. G. The exercise program proceeds to the next exercise. "(...)" indicates that not all screens of the dialogue are shown.

The program investigated was a smartphone-based application that is Conformité Européenne (CE) marked and approved as a medical device directive (MDD) class I medical device. It consists of a series of specific exercises that include a multidimensional progression module. In brief, participants were guided through a pain and functional assessment at baseline and were prompted to provide multiloop feedback (ie, after each exercise, as well as on a weekly and monthly basis) as to whether they could complete the individual exercises presented and whether these exercises caused any complaints. If a complaint, primarily any pain sensation, was reported, the progression module was paused, and the intensity of the exercise program was reassessed. Overall pain score assessments were collected every week, and a follow-up functional assessment was prompted every month. Figure 1 presents a schematic illustration of the baseline assessment (A-C) and the progression module (D-G).

### Statistical Analysis

#### Tests for Pain Reduction

The primary hypothesis test for assessing pain score changes is a nonparametric, 2-sided Skillings-Mack test, which is particularly useful for an unbalanced and incomplete block design or in the presence of missing data due to design or missing at random. For self-reported pain scores, the number of observations for the block, the median of the measurement, and the standard deviation were reported for each Skillings-Mack. We used the Bonferroni correction to control familywise errors and reported corrected alpha levels. A Wilcoxon signed-rank test, a Kruskal-Wallis test, and a 1-way ANOVA were employed for secondary analyses of pain and functional scores. Distributions were assessed using the Bartlett test. Corrections for familywise errors were again performed by using Bonferroni correction.

#### Tests for Functional Scores

A time analysis was not feasible for functional scores, and matched pairs were calculated. Based on a Shapiro-Wilk test, a normal distribution could not be assumed. We used a nonparametric method to analyze the functional scores shown. Consequently, the hypothesis test used was a Wilcoxon signed-rank test, and the IQR is reported. After adjustment for familywise errors using Bonferroni correction, statistical significance was assumed when the probability of a type I error was *P*<.0167.

#### Assessment of Retention

Retention was assessed based on whether participants completed at least 1 exercise and submitted a full pain assessment at predefined thresholds (2 weeks, 4 weeks, 8 weeks, and 12 weeks). We hence report the proportions of the initially included study population.

## Results

### Study Population

As the study population at hand was enrolled prior to the program being subject to a prescription by physicians and other authorized health care providers, the enrollment was primarily based on self-selection through out-of-pocket pay or the use of voucher codes, which were handed out through marketing campaigns over the period of the data collection to evaluate the program at hand. A total of 2517 participants (63% female, mean age 47.08, SD 14.61 years) met the inclusion criteria and provided at least 2 data points necessary for the intraindividual control over 12 weeks. Measurements were collected after 2, 4, 8, and 12 weeks of use. Demographic characteristics on age and sex were collected to investigate differences of age groups in pain duration (ie, acute, subacute, chronic, not specified) and pain area (ie, lower back, upper back, hip, knee). Baseline demographics are displayed in Table 1. At baseline, 1864 (74.06%) patients did not receive physiotherapy in addition to Vivira, while 653 (25.94%) received physiotherapy in addition to Vivira. Moreover, 2023 (80.37%) patients reported at baseline that they did not take any pain medication, while 494 (19.63%) reported that they took pain medication.

**Table 1:** Baseline characteristics of the study population.

Characteristics		Reported pain area					Reported pain duration			
		Lower back, n (%)	Upper back, n (%)	Hip, n (%)	Knee, n (%)	Acute, n (%)		Subacute, n (%)	Chronic, n (%)	Not specified, n (%)
**Age**										
	18-35	312 (24.4)	196, (42.8)	58 (18.6)	148 (31.6)	110 (37.9)		85 (36.3)	243 (26.2)	276 (25.9)
	36-45	255 (20)	71 (15.5)	52 (16.7)	58 (12.4)	59 (20.3)		46 (19.7)	145 (15.6)	186 (17.5)
	46-55	340 (26.6)	118 (25.8)	94 (30.1)	87 (18.6)	69 (23.8)		48 (20.5)	236 (25.4)	286 (26.9)
	56-65	268 (21)	46 (10)	82 (26.3)	126 (26.9)	38 (13.1)		36 (15.4)	230 (24.8)	218 (20.5)
	66-75	82 (6.4)	23 (5)	22 (7.1)	45 (9.6)	13 (4.5)		18 (7.7)	68 (7.3)	73 (6.9)
	75+	15 (1.2)	2 (0.4)	4 (1.3)	4 (0.9)	1 (0.3)		1 (0.4)	6 (0.6)	17 (1.6)
	Not available^a^	6 (0.5)	2 (0.4)	—^b^	1 (0.2)	—		—	—	9 (0.8)
**Sex**										
	Female	770 (57)	318 (69.4)	211 (67.6)	287 (61.2)	182 (62.8)		138 (59)	638 (68.8)	628 (59)
	Male	580 (43)	140 (30.6)	101 (32.4)	182 (38.8)	108 (37.2)		96 (41)	290 (31.3)	437 (41)

^a^Not available because some patients did not provide their age when asked in the initial interaction.

^b^No patients in the population with this specification existed.

### Overall Pain Reduction

**Figure 2:**
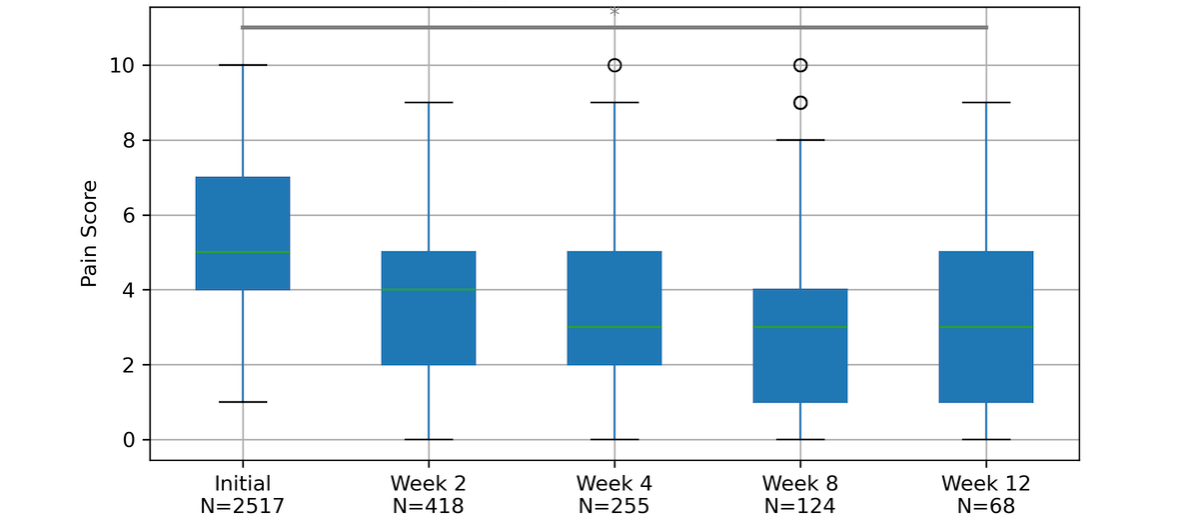
Average self-reported pain score for each retention period for all pain areas. Centerline (green), median; boxplot limits, upper and lower quartiles; whiskers, 1.5x IQR; points, outliers; *P*<.05 =* for the Skillings-Mack Test. Skillings-Mack Test for Initial, Week 2, Week 4, Week 8, Week 12. *T*_2516_=2728.27, *T*<.05

We saw a substantial reduction in self-reported pain scores across 2, 4, 8, and 12 weeks (*t*_2516_=2728.27, *P*=.03). Self-reported pain scores at the start were, on average, 5.19 (SD 1.96) out of 10; after 2 weeks, 3.72 (SD 2.06) out of 10; after 4 weeks, 3.39 (SD 2.35) out of 10, after 8 weeks, 3.19 (SD 2.44) out of 10; and after 12 weeks, 3.35 (SD 2.38) out of 10. These differences are illustrated in Figure 2 and described in Tables 2 and 3.

**Table 2:** Self-reported pain scores and changes across indication subsets and reported pain duration by retained days.

Pain Area	Lower back	Lower back	Lower back	Lower back	Lower back	Upper back	Upper back	Upper back	Upper back	Upper back
Pain duration	All	Acute	Subacute	Chronic	Not specified	All	Acute	Subacute	Chronic	Not specified
Initial, n (%)	1278 (37)	144 (4)	107 (3)	443 (13)	584 (17)	458 (13)	74 (2)	50 (1)	170 (5)	164 (5)
Initial, mean (SD)	5.33 (1.98)	4.47 (1.76)	5 (1.54)	5.37 (1.84)	5.57 (2.13)	5.19 (1.84)	4.57 (1.76)	4.96 (1.74)	5.15 (1.73)	5.57 (1.93)
Week 2, n (%)	202 (36)	30 (5)	26 (5)	120 (21)	26 (5)	81 (14)	15 (3)	15 (3)	47 (8)	4 (1)
Week 2, mean (SD)	3.97 (2.04)	3.23 (2.1)	3.54 (1.73)	4.2 (2.05)	4.19 (2.02)	3.65 (2.11)	3.13 (2.53)	4.27 (2.22)	3.57 (1.93)	—^a^
Week 4, n (%)	119 (36)	16 (5)	17 (5)	69 (21)	17 (5)	46 (14)	11 (3)	4 (1)	26 (8)	5 (2)
Week 4, mean (SD)	3.63 (2.38)	2.19 (1.56)	3.71 (1.99)	4.12 (2.39)	2.94 (2.77)	2.91 (2.03)	3.18 (1.89)	—	2.81 (2)	—
Week 8, n (%)	57 (39)	10 (7)	4 (3)	39 (26)	4 (3)	17 (11)	4 (3)	2 (1)	9 (6)	2 (1)
Week 8, mean (SD)	3.58 (2.41)	2.6 (2.12)	—	4 (2.52)	—	3.65 (2.98)	—	—	3 (2.4)	9 (1.41)
Week 12, n (%)	33 (39)	5 (6)	3 (4)	23 (27)	2 (2)	9 (11)	0 (0)	0 (0)	7 (8)	2 (2)
Week 12, mean (SD)	4.12 (2.63)	2.8 (3.27)	—	4.35 (2.62)	—	3.67 (2.5)	—	—	2.86 (2.04)	—
SM^b^ test value	1361.13	156.39	115.34	523.17	571.83	487.45	—	—	187.02	159.81
SM degrees of freedom	1271	143	106	439	580	457	—	—	169	163
SM adjusted values^c^	0.8	0.9	0.9	0.07	0.9	0.9	—	—	0.9	0.9

^a^No sufficient data was available to calculate the statistics.

^b^SM: Skillings-Mack.

^c^The adjusted *P* values were calculated using Bonferroni corrections.

**Table 3:** Self-reported pain scores and changes across indication subsets and reported pain duration by retained days.

Pain Area	Hip	Hip	Hip	Hip	Hip	Knee	Knee	Knee	Knee	Knee
Pain duration	All	Acute	Subacute	Chronic	Not specified	All	Acute	Subacute	Chronic	Not specified
Initial, n (%)	62 (23)	6 (2)	11 (4)	42 (16)	3 (1)	73 (27)	9 (3)	10 (4)	51 (19)	3 (1)
Initial, mean (SD)	3.87 (2.08)	3 (2)	4.09 (1.97)	3.93 (2.12)	—^a^	2.97 (1.91)	2.22 (1.56)	3.8 (1.32)	3.06 (2.01)	—
Week 2, n (%)	44 (24)	2 (1)	8 (4)	33 (18)	1 (1)	46 (26)	3 (2)	6 (3)	31 (17)	6 (3)
Week 2, mean (SD)	3.93 (2.43)	—	4 (1.51)	3.94 (2.73)	—	2.72 (2.33)	—	1 (0.89)	2.97 (2.24)	4 (3.03)
Week 4, n (%)	23 (23)	2 (2)	4 (4)	17 (17)	—	27 (27)	1 (1)	2 (2)	22 (22)	2 (2)
Week 4, mean (SD)	3.04 (2.46)	0.5 (0.71)	3.75 (2.99)	3.18 (2.38)	—	2.22 (1.91)	—	—	2.41 (2.02)	—
Week 8, n (%)	7 (13)	1 (2)	1 (2)	4 (8)	1 (2)	19 (37)	2 (4)	3 (6)	14 (27)	0 (0)
Week 8, mean (SD)	3.14 (2.04)	—	—	—	—	1.95 (1.18)	—	—	1.93 (1.33)	—
Week 12, n (%)	7 (13)	1 (2)	1 (2)	4 (8)	1 (2)	19 (37)	2 (4)	3 (6)	14 (27)	—
Week 12, mean (SD)	3.14 (2.04)	—	—	2.75 (2.06)	—	1.95 (1.18)	—	—	1.93 (1.33)	—
SM^b^ test value	353.05	—	—	174.65	—	508.86	—	48.58	217.42	—
SM degrees of freedom	311	—	—	137	—	467	—	47	175	—
SM adjusted values^c^	0.9	—	—	0.35	—	0.9	—	0.9	0.34	—

^a^No sufficient data was available to calculate the statistics.

^b^SM: Skillings-Mack.

^c^The adjusted *P* values were calculated using Bonferroni corrections.

### Post Hoc Analysis Comparison of Sequential Data Entry Points

**Figure 3:**
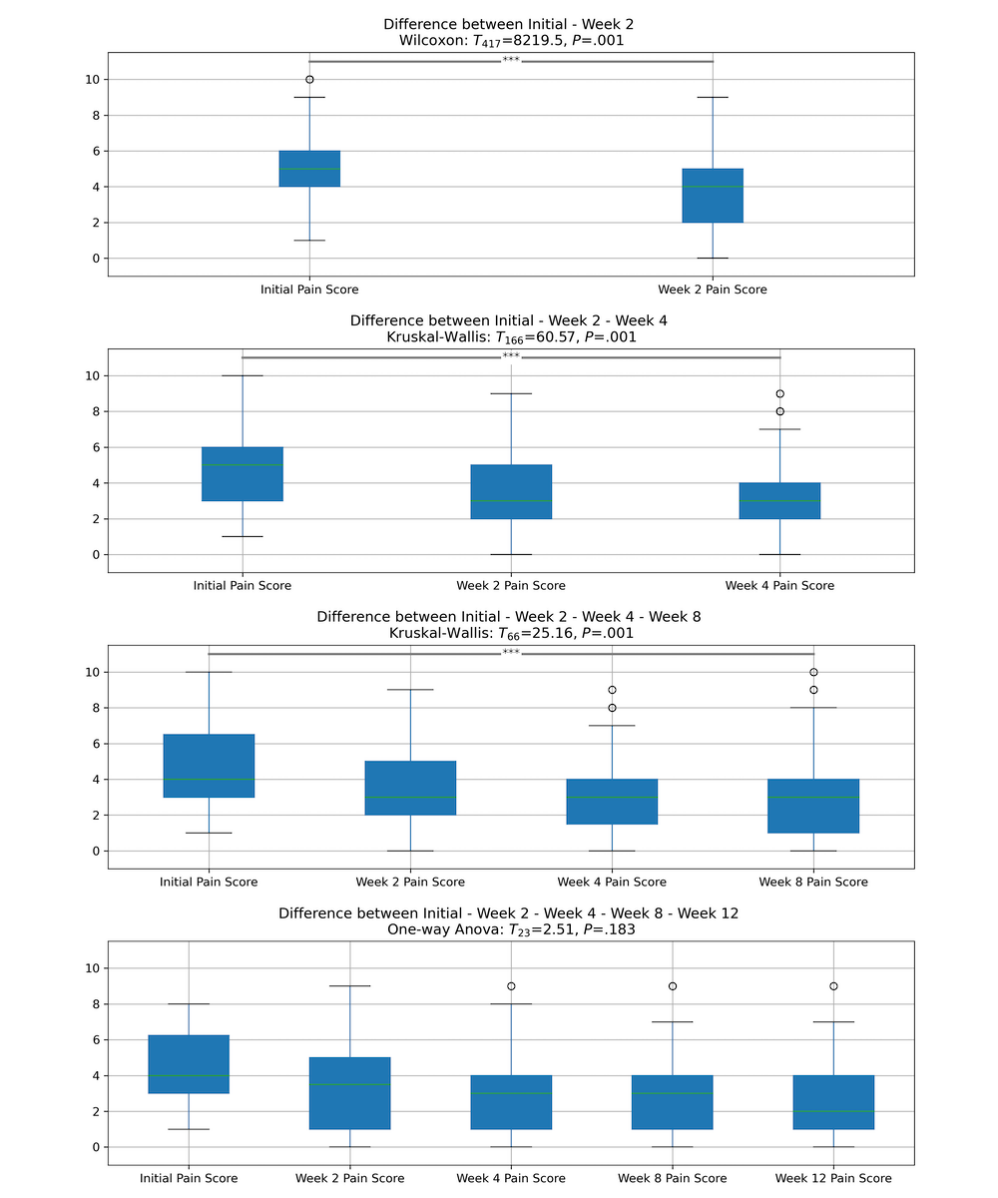
Post hoc results for self-reported pain scores when comparing different assessment times. Centerline (green), median; boxplots limits, upper and lower quartiles; whiskers, 1.5x IQR; points, outliers; *P* values for the Wilcoxon signed-rank test (initial and 2 weeks), the Kruskal-Wallis test (initial, 2 and, 4 weeks and initial, 2, 4, and 8 weeks), and the 1-way ANOVA (initial, 2, 4, 8, and 12 weeks) are displayed on the line.

We calculated further post hoc tests to investigate the effect of different assessment times and, consequently, different durations of exposure to the program. We used the Bonferroni method to adjust for familywise errors. First, we calculated a Wilcoxon signed-rank test to investigate to what degree a change in pain reduction occurred in participants that provided self-reported data at the initial assessment and after using the home exercise program for 2 weeks. We found a significant difference between the initial assessment (median 5) and the assessment after 2 weeks (median 4; *t*_417_=8219.5, *P*<.001). Second, we calculated a Kruskal-Wallis test showing that the self-reported pain values differed significantly between the initial (median 5), 2-week (median 3), and 4-week assessments (median 3; *t*_166_=60.56, *P*<.001). Third, we calculated a Kruskal-Wallis test showing that the self-reported pain values differed significantly between the initial (median 4), 2-week (median 3), 4-week (median 3), and 8-week assessments (median 3, *t*_66_=25.16, *P*<.001). Finally, as this subsample was normally distributed and had an equal variance as indicated by Bartlett test, we calculated a 1-way ANOVA showing a nonsignificant difference in self-reported pain for the initial (mean 4.62, SD 2.12), 2-week (mean 3.5, SD 2.39), 4-week (mean 3.2, SD 2.48), 8-week (mean 2.8, SD 2.44), and 12-week (mean 2.66, SD 2.51) assessments (*F*_23_=2.51, *P*=.18). Figure 3 illustrates these findings and highlights that, given the retention outlined below, shorter exercise periods also showed an overall clinical outcome on pain score reduction. Finally, we investigated whether the initial pain score differed for patients completing the intervention (providing a final data point after 12 weeks) and for patients who did not complete the intervention. Using a Mann-Whitney *U* rank test, we found no significant difference between the reported initial pain of the group providing a data point after 12 weeks (n=68, median 5) and the group providing no data point after 12 weeks (n=2449, median 5; *U*=79470, *P*=.516).

### Stratum-Specific Changes in Pain Intensity

After stratifying the available data for pain area and pain duration as a secondary analysis, we saw a comparable response pattern across all pain areas. Participants with lower back pain reported a reduction in their initial pain score from 5.33 to 4.12 after 12 weeks of exercises (*t*_1271_=1361.13, *P*=.80). The subpopulation of participants with chronic lower back pain saw a marked improvement from 5.37 to 4.35 (*t*_439_=523.17, *P*=.07). Similarly, participants with upper back pain reported a reduction of their pain intensity from 5.19 to 3.67 after completing the exercise program (*t*_457_=478.45, *P*=.90). The pain score change in participants with hip pain was on a comparable trajectory; we saw a reduction from a baseline pain score of 5.21 to 3.14 after 12 weeks (*t*_311_=353.05, *P*=.90). Finally, participants with knee pain saw an improvement from a baseline of 4.8 to 1.95 after completing the exercise program (*t*_467_=508,86, *P*=.90). As the employed Skillings-Mack test cannot provide values for lacking blocks, no substratum analyses for acute and subacute upper back pain, acute, subacute, and nonspecified hip pain, and acute and nonspecified knee pain could be reported (Tables 2-3, Multimedia Appendix 1).

### Functional Scores

Another secondary outcome was to assess the improvement of a set of functional scores. The lower and upper back showed significant improvement in strength and mobility and total functional score (Table 4). This finding is consistent with overall intervals of available submitted scores studied, except for the upper back, which did not have a significantly improved strength score between participants’ first and fourth submissions (Table 5). For coordination, the upper back and lower back did not show significant improvement across any intervals of submitted scores studied, except for the upper back between the first and fourth submissions of functional scores, where a significant improvement in coordination score was observed (Table 6). The knee and hip showed a significant improvement in mobility (Table 5) and coordination (Table 6), as well as total functional (Table 3) score between the first and second submission of functional scores. However, they did not show a significant improvement in strength across any completed submission (Table 3). For the hip and knee, no significant improvement could be shown for mobility (Table 5), coordination (Table 6), and total functional score (Table 3) could be shown between the first and third and first and fourth submissions of functional scores.

For coordination, the upper back and lower back did not show significant improvement across any intervals of submitted scores studied, except for the upper back between the first and fourth submission of functional scores, where a significant improvement in coordination score was observed (Table 6). The knee and hip showed a significant improvement in mobility (Table 6) and coordination (Table 7), as well as total functional (Table 4) score between the first and second submission of functional scores. However, they did not show a significant improvement in strength across any completed submission (Table 4). For the hip and knee, no significant improvement was shown for mobility (Table 6), coordination (Table 7), and total functional score (Table 4) between the first and third and first and fourth submissions of functional scores.

**Table 4:** Total functional score for matched comparison and pain area.

Matched comparison and pain area			Pain area, n		Retained days, median (IQR)		Initial, median (IQR)		Last, median (IQR)		Test
**First and second entry**											
	Lower back	132		29 (20.5-38.5)		60 (43-75)		71.5 (53-81.5)		*P*<.001	
	Upper back	38		29 (20.5-38.5)		65 (43-80)		71.5 (60-83)		*P*<.001	
	Hip	40		29 (20.5-38.5)		67 (43-77)		70 (55-80)		*P*<.05	
	Knee	47		29 (20.5-38.5)		70 (50-83)		80 (57-87)		*P*<.01	
**First and third entry**											
	Lower back	48		59 (48-80)		60 (43-75)		78.5 (60-87)		*P*<.001	
	Upper back	15		59 (48-80)		65 (43-80)		73 (63-87)		*P*<.05	
	Hip	16		59 (48-80)		67 (43-77)		60 (41.5-81.5)		*P*=.0525	
	Knee	20		59 (48-80)		70 (50-83)		80 (73-83)		*P*=.0348^a^	
**First and fourth entry**											
	Lower back	25		88.5 (72-112)		60 (43-75)		80 (67-87)		*P*<.05	
	Upper back	8		88.5 (72-112)		65 (43-80)		81.5 (67-96.5)		*P*<.05	
	Hip	5		88.5 (72-112)		67 (43-77)		67 (63-80)		*P*=.0625	
	Knee	13		88.5 (72-112)		70 (50-83)		80 (73-87)		*P*=.1592	

^a^Due to adjustments to the *P* level (Bonferroni correction), these values are not significant.

**Table 5:** Strength functional score for matched comparison and pain area.

Matched comparison and pain area			Pain area, n		Retained days, median (IQR)		Initial, median (IQR)		Last, median (IQR)		Test
**First and second entry**											
	Lower back	132		29 (20.5-38.5)		60 (30-80)		70 (40-100)		*P*<.001	
	Upper back	38		29 (20.5-38.5)		60 (40-80)		70 (60-100)		*P*<.05	
	Hip	40		29 (20.5-38.5)		60 (40-100)		80 (55-100)		*P*=.0213^a^	
	Knee	47		29 (20.5-38.5)		70 (50-90)		80 (60-100)		*P*=.0249^a^	
**First and third entry**											
	Lower back	48		59 (48-80)		60 (30-80)		80 (60-100)		*P*<.001	
	Upper back	15		59 (48-80)		60 (40-80)		80 (60-100)		*P*<.05	
	Hip	16		59 (48-80)		60 (40-100)		60 (45-95)		*P*=.0498^a^	
	Knee	20		59 (48-80)		70 (50-90)		80 (60-100)		*P*=.0797	
**First and fourth entry**											
	Lower back	25		88.5 (72-112)		60 (30-80)		80 (60-100)		*P*<.05	
	Upper back	8		88.5 (72-112)		60 (40-80)		80 (50-100)		*P*=.1250	
	Hip	5		88.5 (72-112)		60 (40-100)		60 (60-80)		*P*=.3125	
	Knee	13		88.5 (72-112)		70 (50-90)		90 (60-100)		*P*=.0938	

^a^Due to adjustments to the *P* level (Bonferroni correction), these values are not significant.

**Table 6:** Mobility functional score for matched comparison and pain area.

Matched comparison and pain area			Pain area, n		Retained days, median (IQR)	Initial, median (IQR)	Last, median (IQR)		Test
**First and second entry**									
	Lower back	132		29 (20.5-38.5)		60 (47.5-80)	70 (55-80)	*P*<.001	
	Upper back	38		29 (20.5-38.5)		62.5 (50-75)	70 (60-90)	*P*<.001	
	Hip	40		29 (20.5-38.5)		60 (45-77.5)	70 (50-80)	*P*<.05	
	Knee	47		29 (20.5-38.5)		60 (50-80)	70 (55-85)	*P*<.01	
**First and third entry**									
	Lower back	48		59 (48-80)		60 (47.5-80)	75 (60-85)	*P*<.01	
	Upper back	15		59 (48-80)		62.5 (50-75)	75 (60-90)	*P*<.05	
	Hip	16		59 (48-80)		60 (45-77.5)	65 (40-82.5)	*P*=.1187	
	Knee	20		59 (48-80)		60 (50-80)	80 (72.5-82.5)	*P*=.1191	
**First and fourth entry**									
	Lower back	25		88.5 (72-112)		60 (47.5-80)	70 (65-90)	*P*<.05	
	Upper back	8		88.5 (72-112)		62.5 (50-75)	82.5 (75-95)	*P*<.05	
	Hip	5		88.5 (72-112)		60 (45-77.5)	70 (65-70)	*P*=.0625	
	Knee	13		88.5 (72-112)		60 (50-80)	80 (70-85)	*P*=.2695	

**Table 7:** Coordination functional score for matched comparison and pain area.

Matched comparison and pain area			Pain area, n	Retained days, median (IQR)		Initial, median (IQR)		Last, median (IQR)		Test
**First and second entry**										
	Lower back	132		29 (20.5-38.5)	70 (40-80)		80 (55-80)		*P*=.2806	
	Upper back	38		29 (20.5-38.5)	80 (50-80)		80 (60-100)		*P*=.0585	
	Hip	40		29 (20.5-38.5)	60 (35-80)		80 (50-85)		*P*<.05	
	Knee	47		29 (20.5-38.5)	60 (40-80)		70 (50-80)		*P*<.05	
**First and third entry**										
	Lower back	48		59 (48-80)	70 (40-80)		80 (60-100)		*P*=.2187	
	Upper back	15		59 (48-80)	80 (50-80)		80 (60-100)		*P*<.05	
	Hip	16		59 (48-80)	60 (35-80)		70 (50-90)		*P*=.1717	
	Knee	20		59 (48-80)	60 (40-80)		80 (60-80)		*P*=.0885	
**First and fourth entry**										
	Lower back	25		88.5 (72-112)	70 (40-80)		80 (80-80)		*P*=.6509	
	Upper back	8		88.5 (72-112)	80 (50-80)		80 (60-100)		*P*=.0938	
	Hip	5		88.5 (72-112)	60 (35. 80)		80 (60-100)		*P*=.0625	
	Knee	13		88.5 (72-112)	60 (40-80)		80 (70-80)		*P*=.2422	

### Retention

**Figure 4:**
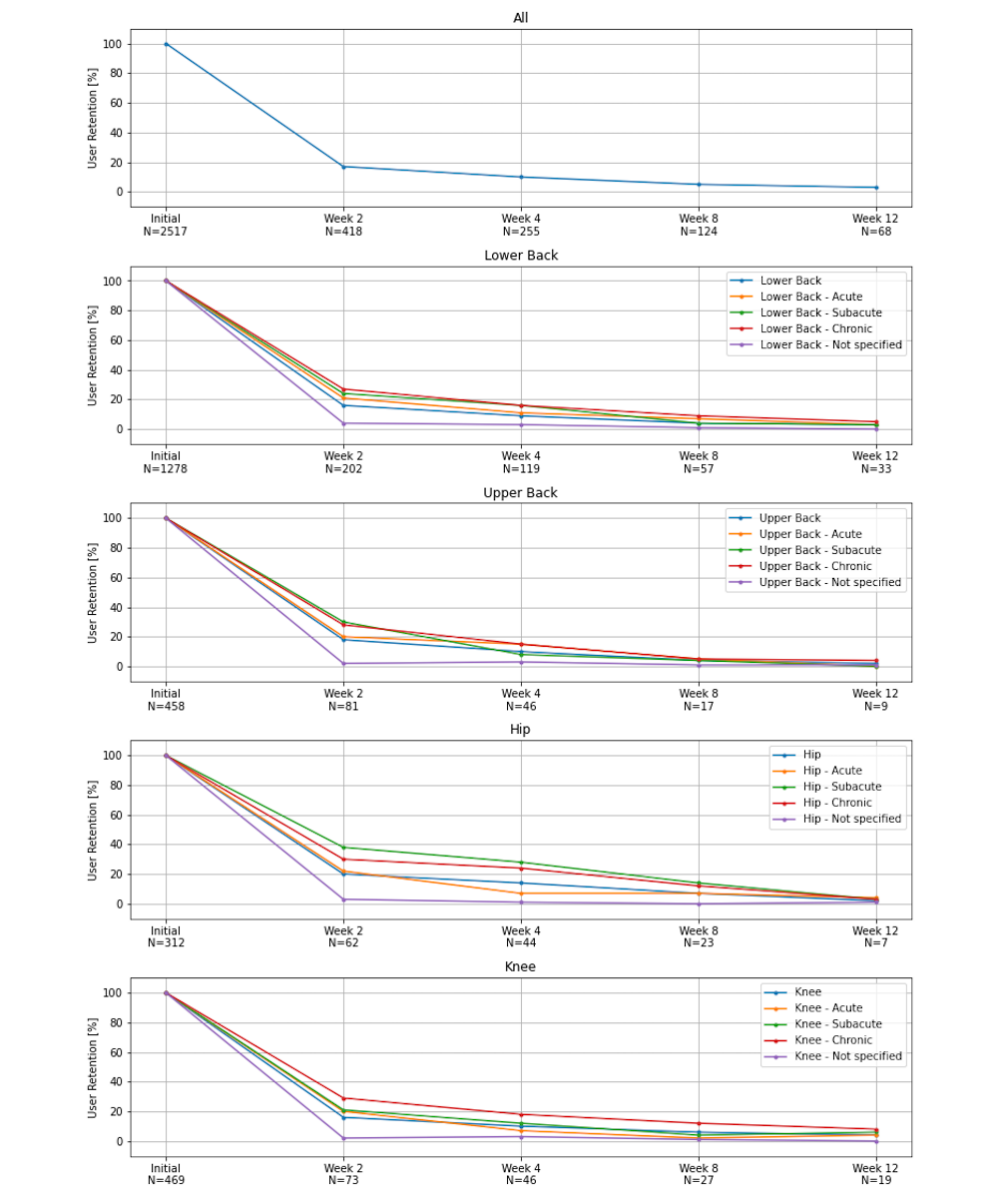
Retention rate for different pain areas and durations.

As a third secondary analysis, the retention rate for the program at hand was examined. The overall retention rate was 17% after 2 weeks, 10% after 4 weeks, 4% after 8 weeks, and 3% after 12 weeks (Table S1 in Multimedia Appendix 1). This high attrition was present in all subpopulations, and no difference in the loss to follow-up patterns could be detected. However, total attrition could be observed in participants with pain in the lower back and nonspecified pain duration, upper back with acute and subacute pain durations, and knee with a nonspecified pain duration (Table S1 in Multimedia Appendix 1). Nonetheless, we noticed a tendency toward higher retention rates among participants with chronic pain (Figure 4).

## Discussion

### A Digital Home Exercise Program Can Lead to Significant Improvements in Pain Scores

Because exercise is known to effectively address unspecific and degenerative musculoskeletal pain [4-6], a digitally guided home exercise program was a priori considered a practical therapeutic intervention to address this spectrum of conditions. The overall analysis of the data set supports this assumption and shows a significant improvement in self-reported pain scores based on a VNRS (Figure 2, Table 2). Although the presented observational data do not yield confirmatory power, we consider the improvement of self-reported pain scores an effect of the home exercise treatment and not an indicator of spontaneous improvement. This consideration is based on prior research demonstrating a lower-than-expected rate of spontaneous improvement for MSP in general and for back pain in particular [17]. These findings were particularly emphasized in participants with established or chronic pain [18]. Hence, participants with chronic pain were greatly overrepresented in our study population, at 36.9% (n=928) at baseline and 70.6% (n=48) after 12 weeks of follow-up, compared to an expected prevalence of chronic back pain of 15.5% in the source population [19], so we deem this interpretation applicable. Additional post hoc analyses showed significant improvements between the initial and 2-week assessments, the initial, 2-week, and 4-week assessments, and the initial, 2-week, 4-week, and 8-week assessments. Yet, they failed to show significant improvements between all assessment time points (Figure 4). We conclude from these analyses that an indicator for an overall improvement in pain scores is given and that shorter periods of exposure to the home exercise program yielded significant pain score improvements over the abbreviated time points (ie, up until 8 weeks). Nevertheless, conclusions based on this data set warrant careful interpretation, as a high attrition rate is prone to bias.

### Secondary Analyses of Subpopulations Did Not Yield Relevant Pain Score Reductions

An exploratory stratification across different pain areas (ie, upper back, lower back, hip, and knee) and different pain durations (ie, acute, subacute, and chronic pain) did not significantly improve the pain scores reported. However, repeated corrections for familywise errors were required to perform this analysis correctly. Therefore, a significantly lower alpha level had to be applied. From the insignificant improvements, however, we saw a tendency toward a relevant improvement in pain scores for lower back *(P=*.039), hip *(P=*.05), and knee *(P=*.088). These data suggest a more nuanced response to a home exercise program across different pain areas. However, the available data did not provide a sufficient density to investigate this issue thoroughly.

### Functional Improvements Showed a Differential Pattern

Except for hip and knee, significant improvements in strength and mobility could be detected between the first and the second assessment of the functional ability. However, participants with hip and knee pain showed a significant response in terms of increasing their coordination. This indicates a secondary benefit of the examined program. Interestingly, participants with lower back pain showed a particularly sustained response over an extended period (median follow-up of 88.5 days, IQR 72-112) in the dimensions of strength and mobility. We interpret this as an indicator of a differential functional response to the respective exercise programs. Because the transformation of the functional test results (ie, the test could be completed successfully or the test could not be completed successfully) into a discrete score (ie, mobility, strength, coordination, and total score) was solely based on expert consensus, a thorough validation of the assessment is required. Therefore, a careful interpretation of these results is warranted because of the limited data availability.

### Retention Rates Were Within the Expected Range of a Digital Therapeutic

Retention rates to digital therapeutics have proven to show both high attrition to use and attrition to follow-up. For example, Baumel et al [20] reported an average adherence to mental health digital therapeutics of <10% after 30 days of use. Similarly, Fleming et al [21] presented a systematic review on the intensity of digital therapeutics use in mental health and reported a sustained use (ie, completion of a program or continuation for more than 6 weeks) between 0.5% and 28.6%. The retention rates in this study were within this spectrum; only the spectrum of hip pain reached a retention rate of 14% after 4 weeks and exceeded the expected range. After 12 weeks (ie, upon completion of the exercise program), an average retention rate of 3% was demonstrated.

The low retention to digital therapeutics demonstrates a key challenge for evaluation, as insufficiently reported outcome data limit the interpretability of the clinical outcomes obtained. This circumstance mandates further research on how participant behavior (ie, continuation or discontinuation of the exercise regime as prompted) relates to retention to a study and, consequently, the clinical value of digital therapeutics.

### Limitations

Because this study was based on participant-initiated enrollment and self-reported data, a number of limitations need to be discussed. Regarding the study population, we saw an overrepresentation of female participants. Comparable studies have presented similar sex distributions when allowing for self-selection of participation but have not concluded on the potential implications of this imbalance. A potential, nonexhaustive explanation could lie in the differential awareness of health and information–seeking behavior for health-related questions, which favors women to discover and adopt offered health care services more quickly [22]. Additionally, participants with chronic pain were overrepresented in our study population. This leads to our understanding that the therapeutic effects observed were plausibly due to the program examined and not due to the natural course of the spectrum of conditions studied.

Nonetheless, the drivers and potential implications of this imbalance remain unclear. In addition, the self-assessed and self-reported outcome data are subject to a certain interindividual difference. However, the VNRS employed has been shown to be particularly applicable in a day-to-day setting [10], valid [9,23], and reliable [9]. This, however, does not apply to the functional assessments employed. Although all assessments were based on a set of validated orthopedic tests, the transformation of the binary assessment into a discrete scale, as outlined earlier in this report, has only been validated through an expert panel review and lacks quantitative validation. Overall, we see a valid indication for a therapeutic benefit of the program assessed but acknowledge that the presented data warrant a careful interpretation.

### Comparison With Prior Work

The clinical outcomes of interventions in general (ie, without a key digital component) to improve PA was reviewed in a meta-analysis showing no significant short-term, intermediate, or long-term improvements [8]. Studies focusing specifically on digital health interventions have been reviewed in different studies. One systematic review investigating the adherence to digital interventions aiming to increase PA in patients with MSP showed no significant difference in adherence to exercises between conventional and therapeutic exercises (standardized mean difference 0.23, 95% CI –0.10 to 0.57) [24]. Another systematic review focusing on digital health interventions' clinical outcomes addressing MSCs showed significantly better results for digital therapeutics than the control [25]. Two studies [26,27] included in the review had a similar focus to this work. However, these studies were randomized controlled trials and did not investigate clinical outcomes in a real-world setting.

### Conclusions

Innovative therapeutic means are required to address the increasing burden of disease from MSCs. This study presents early observational use data on the clinical outcomes of a program in terms of overall self-reported pain score reduction and demonstrates significant improvement in its primary analysis. However, stratum-specific pain reductions did not reach the adjusted level of significance. Significant functional improvements, particularly in strength and mobility, could be demonstrated for upper and lower back pain but not for hip and knee pain. Nevertheless, coordination improved significantly in participants with hip and knee pain.

Interestingly, chronic back pain profited from the extended use and showed significant increases in strength and mobility scores after a median of 88.5 days. Retention was shown to be low but was within the spectrum of what the available literature allows us to expect. Further research is required to substantiate the early indicators of the examined program's therapeutic benefit and quantify the clinical relevance of the improvements achieved.

## Appendix

Multimedia Appendix 1 Further figure regarding pain scores and table displaying adherence.

## Abbreviations

CE: Conformité Européenne
MDD: medical device directive
MSC: musculoskeletal condition
MSP: musculoskeletal pain
PA: physical activity
VNRS: verbal-numerical rating scale
