# Supplementary material for: Preliminary Use and Outcome Data of a Digital Home Exercise Program for Back, Hip, and Knee Pain: Retrospective Observational Study With a Time Series and Matched Analysis
Source: JMIR Mhealth Uhealth. 2022 Dec 2;10(12):e38649. doi: 10.2196/38649 (PMC9758631; doi:10.2196/38649)
Supplement: Multimedia Appendix 1 [file mhealth_v10i12e38649_app1.docx]

## Multimedia Appendix 1

**Supplementary Figure 1:** Average self-reported pain score for each retention period for all pain areas. Centerline (green), median; boxplot limits, upper and lower quartiles; whiskers, 1.5x interquartile range; points, outliers; *P* values for the Skillings-Mack Test are given in the heading of each subfigure, 'nan’ = not a number, null value.


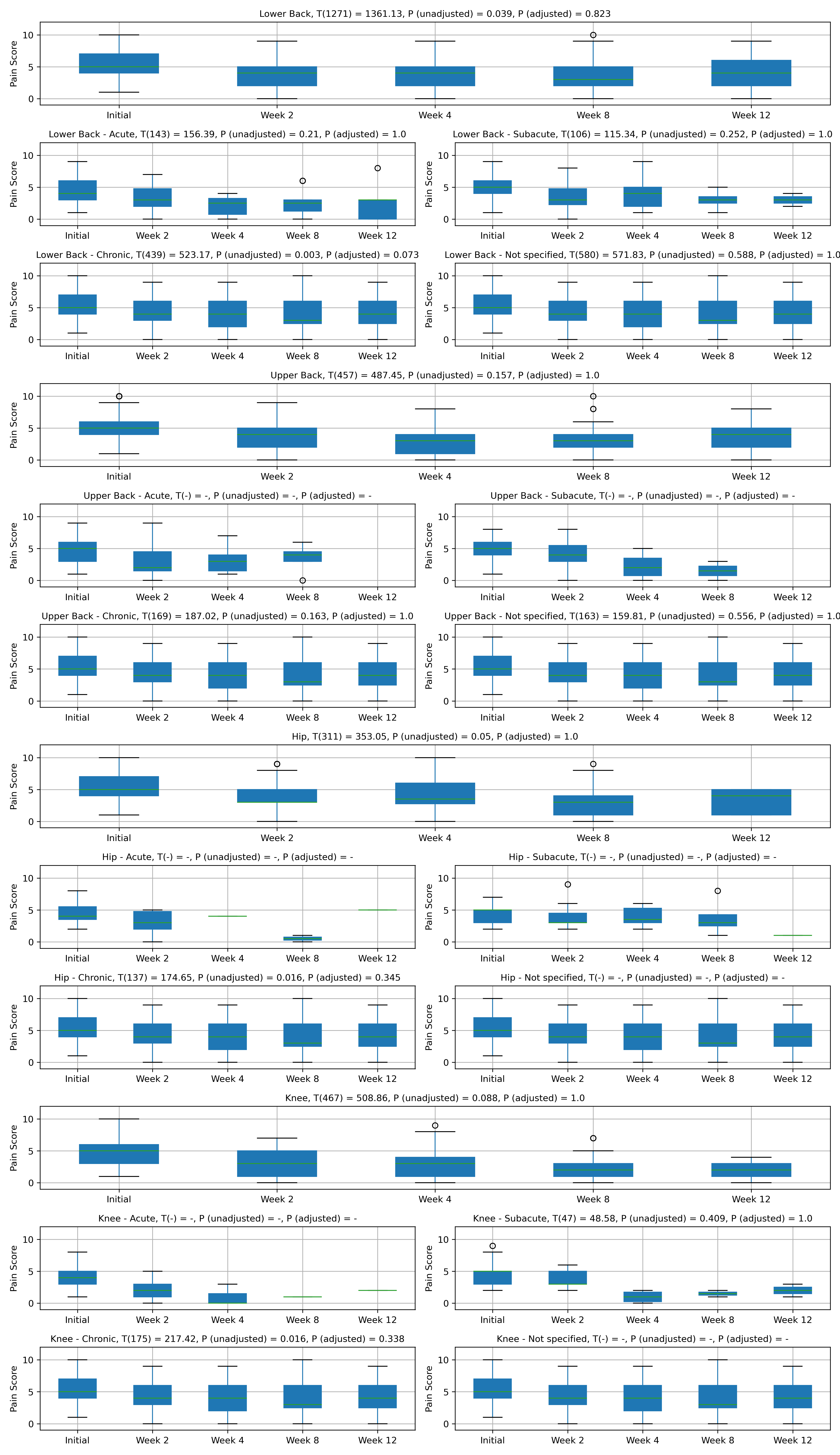


Supplementary Table 1. User Retention across indication subsets and reported pain duration.

| **Indication**  **Subset** | **Pain Duration** | **Initial**  **(N, %)** | **Week 2**  **(N, %)** | **Week 4**  **(N, %)** | **Week 8**  **(N, %)** | **Week 12**  **(N, %)** |
| --- | --- | --- | --- | --- | --- | --- |
| **All** | All | 2517(100%) | 418(17%) | 255(10%) | 107(4%) | 68(3%) |
| **Lower Back** | All | 1278(100%) | 202(16%) | 119(9%) | 57(4%) | 33(3%) |
|  | Acute | 144(100%) | 30(21%) | 16(11%) | 10(7%) | 5(3%) |
|  | Subacute | 107(100%) | 26(24%) | 17(16%) | 4(4%) | 3(3%) |
|  | Chronic | 443(100%) | 120(27%) | 69(16%) | 39(9%) | 23(5%) |
|  | Not specified | 584(100%) | 26(4%) | 17(3%) | 4(1%) | 2(1%) |
| **Upper Back** | All | 458(100%) | 81(18%) | 46(10%) | 17(4%) | 9(2%) |
|  | Acute | 74(100%) | 15(20%) | 11(15%) | 4(5%) | 0(0%) |
|  | Subacute | 50(100%) | 15(30%) | 4(8%) | 2(4%) | 0(0%) |
|  | Chronic | 170(100%) | 47(28%) | 26(15%) | 9(5%) | 7(4%) |
|  | Not specified | 164(100%) | 4(2%) | 5(3%) | 2(1%) | 2(1%) |
| **Hip** | All | 312(100%) | 62(20%) | 44(14%) | 23(7%) | 7(2%) |
|  | Acute | 27(100%) | 6(22%) | 2(7%) | 2(7%) | 1(4%) |
|  | Subacute | 29(100%) | 11(38%) | 8(28%) | 4(14%) | 1(3%) |
|  | Chronic | 138(100%) | 42(30%) | 33(24%) | 17(12%) | 4(3%) |
|  | Not specified | 118(100%) | 3(3%) | 1(1%) | 0(0%) | 1(1%) |
| **Knee** | All | 469(100%) | 73(16%) | 46(10%) | 27(6%) | 19(4%) |
|  | Acute | 45(100%) | 9(20%) | 3(7%) | 1(2%) | 2(4%) |
|  | Subacute | 48(100%) | 10(21%) | 6(12%) | 2(4%) | 3(6%) |
|  | Chronic | 177(100%) | 51(29%) | 31(18%) | 22(12%) | 14(8%) |
|  | Not specified | 199(100%) | 3(2%) | 6(3%) | 2(1%) | 0(0%) |
